# UV radiation and temperature increase alter the PSII function and defense mechanisms in a bloom-forming cyanobacterium *Microcystis aeruginosa*

**DOI:** 10.3389/fmicb.2024.1351796

**Published:** 2024-01-16

**Authors:** Fang Yan, Mingze Li, Shasha Zang, Zhiguang Xu, Menglin Bao, Hongyan Wu

**Affiliations:** ^1^School of Life Science, Ludong University, Yantai, China; ^2^Key Laboratory of Marine Biotechnology in Universities of Shandong, Ludong University, Yantai, China

**Keywords:** *Microcystis aeruginosa*, UV radiation, temperature, photosynthesis, cyanobacteria

## Abstract

The aim was to determine the response of a bloom-forming *Microcystis aeruginosa* to climatic changes. Cultures of *M. aeruginosa* FACHB 905 were grown at two temperatures (25°C, 30°C) and exposed to high photosynthetically active radiation (PAR: 400–700 nm) alone or combined with UVR (PAR + UVR: 295–700 nm) for specified times. It was found that increased temperature enhanced *M. aeruginosa* sensitivity to both PAR and PAR + UVR as shown by reduced PSII quantum yields (*F_v_/F_m_*) in comparison with that at growth temperature (25°C), the presence of UVR significantly exacerbated the photoinhibition. *M. aeruginosa* cells grown at high temperature exhibited lower PSII repair rate (*K_rec_*) and sustained nonphotochemical quenching (NPQs) induction during the radiation exposure, particularly for PAR + UVR. Although high temperature alone or worked with UVR induced higher SOD and CAT activity and promoted the removal rate of PsbA, it seemed not enough to prevent the damage effect from them showing by the increased value of photoinactivation rate constant (*K_pi_*). In addition, the energetic cost of microcystin synthesis at high temperature probably led to reduced materials and energy available for PsbA turnover, thus may partly account for the lower *K_rec_* and the declination of photosynthetic activity in cells following PAR and PAR + UVR exposure. Our findings suggest that increased temperature modulates the sensitivity of *M. aeruginosa* to UVR by affecting the PSII repair and defense capacity, thus influencing competitiveness and abundance in the future water environment.

## 1 Introduction

With the process of modern industrialization, nitrogen and other elements such as phosphorus and potassium are increasingly enriched in oceans, lakes and rivers. This enrichment leads to large-scale reproduction of certain species of cyanobacteria in water bodies, resulting in blooms that threaten the quality and sustainability of aquatic ecosystems (He et al., 2016; Zhou et al., 2023). Meanwhile, as a result of the increase in CO_2_ concentration and the depletion of the ozone layer, there has been a gradual rise in both air temperature and ground-level UV radiation, especially UV-B (280–315 nm) (Häder and Barnes, 2019; Williamson et al., 2019). It shows that the global surface temperature increased by 1.1°C in 2011–2020 compared to 1850–1900 with the increasing emissions of greenhouse gas (IPCC, 2023). Increasing temperature may stimulate the development of cyanobacterial blooms by prolonging the growing season, increasing stability of water column and enhancing the growth rates (Visser et al., 2016; Wang et al., 2020; Ma and Wang, 2021). UV radiation usually has negative effects on photosynthesis and growth of cyanobacteria (Rastogi et al., 2014; El-Sheekh et al., 2021). Freshwater ecosystems, particularly in low and mid latitudes, are more likely to be influenced by alterations in UV radiation, as global warming and release of trace gases may reduce the barrier properties of the ozone layer (Williamson et al., 2019). Thus, the combined influence of UV radiation and temperature on algae, particularly cyanobacteria that form blooms on water surfaces, is of significant interest.

*Microcystis aeruginosa* is a bloom-forming cyanobacterium found throughout the world (Huo et al., 2021; Zhang et al., 2023), with several strains producing toxins known as microcystins (MCs) that can lead to poisoning of both humans and livestock. *M. aeruginosa* grows optimally at temperatures between 20 and 25°C (Tan, 2011; Yang et al., 2020; Ji et al., 2021; Guo et al., 2023) and changes in temperature can affect growth and photosynthesis either positively or negatively (Ma et al., 2015; Islam and Beardall, 2020; Zheng et al., 2020; Guo et al., 2023). For example, Zheng et al. found that high temperature at 30 and 35°C in contrast to at 25°C reduced the photosynthetic pigment content and PSII efficiency by increasing reactive oxygen species (ROS) level, thus resulted in growth inhibition in *M. aeruginosa* FACHB 912 (Zheng et al., 2020), similar low growth rate at 30°C was also shown in *M. aeruginosa* CS558 (Islam and Beardall, 2020), in contrast, rapid cell density accumulation from 26°C to 35°C was reported in *M. aeruginosa* FACHB 1343 (Guo et al., 2023), and temperature ≥ 25°C promoted the growth of both strains of *M. aeruginosa* FACHB905 and 419 (Ma et al., 2015). As for UV radiation, its negative influences on *M. aeruginosa* has been demonstrated by numerous studies. UV radiation inhibits the photosynthetic activity (Jiang and Qiu, 2011; Yang et al., 2015), regulates the microcystin synthesis (Ding et al., 2013) and decreases the pigments content, growth rate and abundance of *M. aeruginosa* (Yang et al., 2014; Hernando et al., 2018; De la Rosa et al., 2021).

There have been several recent studies on interactions between these factors. For example, it is reported that UV-B radiation and high temperature led to lower growth rate and photosynthetic pigments content in *M. aeruginosa*, while the activities of antioxidative enzymes were enhanced and production of photoprotective mycosporine-like amino acids (MAAs) was induced (Babele et al., 2017; Cordeiro-Araύjo et al., 2022); in addition, polyunsaturated fatty acids (PUFAs) (especially ω6) are involved in adaptations to increased temperature and UVR stress (De la Rosa et al., 2021). In contrast, raised temperatures have also been reported to counteract the negative influence of UV radiation on growth and photosynthesis in *M. aeruginosa* (Islam and Beardall, 2020; Noyma et al., 2021). Although these results are varied depending on the strain selection or the UV radiation treatments design, the mechanisms responsible for these changes in sensitivity to UV radiation with temperature elevation are not known.

Here, the photosynthetic performance of *M. aeruginosa* under varying temperature and photosynthetically active radiation (PAR) and PAR + UVR conditions were investigated, together with the turnover of PsbA subunits, antioxidant functions, dissipation of excess energy dissipation, and MCs production. The objective was to determine the effects of temperature increases on the UVR sensitivity of *M. aeruginosa,* with specific reference to PSII repair and the defense response.

## 2 Materials and methods

### 2.1 Culture conditions

The toxic FACHB 905 strain of the unicellular cyanobacterium *M. aeruginosa* was obtained from the Institute of Hydrobiology, Chinese Academy of Sciences, and grown in BG-11 medium at 25°C with a 40 μmol m^−2^ s^−1^ (8.2 Wm^−2^) PAR in a photoperiod 12 L:12D in a temperature- and light-controlled incubator (GXZ-280B, Ningbo Jiangnan Instrument, China). According to the summer high temperature at Lake Taihu, China surface water (more than 30°C) (Jiang et al., 2009; Zheng et al., 2020), the culture temperature was set at 25°C for control temperature and 30°C for higher temperature group. The organisms were, respectively, pre-adapted for 7 days at two temperature conditions prior to the radiation treatments. Cultures in the exponential growth phase were used for all experiments.

### 2.2 Radiation treatments

Exponentially growing *M. aeruginosa* cultures were placed in UV-transparent quartz tubes (inside diameter, 6.4 cm; length, 20 cm) with a concentration of about 5× 10^6^ cells mL^−1^ and growth in a temperature-controlled (25 ± 1°C or 30 ± 1°C) flow-through water baths. To assess PSII damage, an inhibitor of chloroplast protein synthesis, lincomycin, was introduced into one of the tubes, with a final concentration of 500 μg mL^−1^. The cultures were grown under aeration (0.3 L min^−1^) with simultaneous exposure to two irradiation treatments, namely, (1) PAR (400–700 nm), with covering of the quartz tubes by Ultraphan film 395 (UV Opak, Digefra, Munich, Germany) and (2) PAR + UVR (295–700 nm), with covering of the tubes by Ultraphan film 295. Radiation was generated by a solar simulator (Sol 1,200, Dr. Hönle GmbH, Germany) with a 1,000-W xenon arc lamp, and irradiance levels were monitored using a radiometer (PMA 2100, Solar light, USA), using 40.8 Wm^−2^ PAR (200 μmol m^−2^ s^−1^) and 5.0 Wm^−2^ UVR, as described (Xu et al., 2019). Cultures were exposed to PAR or PAR + UVR for 90 min and then shifted to growth light of 40 μmol m^−2^ s^−1^ of PAR for 1 h recovery. The cultures were sampled at intervals of 30 min to analyze antioxidant functions, chlorophyll fluorescence, and protein levels using western blotting.

### 2.3 Measurement of chlorophyll fluorescence

A pulse amplitude-modulated fluorometer (WATER-ED PAM, Walz, Germany) was used for measuring chlorophyll fluorescence. The PSII photochemical efficiency (*F_v_/F_m_*) was determined using the minimal (*F_0_*) and maximal (*F_m_*) fluorescence yields of cells that had been adapted to the dark for 10 min, using the formula *F_v_* = (*F_m_*–*F_0_*). For determination of *F_0_* and *F_m_,* low irradiance with 0.5-s pulses of saturating light at <0.1 μmol m^−2^ s^−1^ and 4,000 μmol m^−2^ s^−1^, respectively, were used. A sustained NPQ phase, indicating the inducible increased relaxation time as a fraction of NPQ, persisted following the 10-min period in the dark (Wu et al., 2012). This was defined as NPQs = (*F_mt0_*–*F_m_*)/*F_m_*, where *F_mt0_* indicates the *F_m_* value of samples at time zero prior to radiation exposure, with *F_m_* determined at each specific time point.

Rate constants for photoinactivation (*K_pi_*, s^−1^) and PSII repair (*K_rec_*, s^−1^) were obtained by fitting a single-phase exponential decay curve to the *F_v_/F_m_* versus cumulative photon plot generated during radiation exposure processing of lincomycin-treated samples. *K_rec_* was determined from non-lincomycin treated samples using the Kok equation (Kok, 1956; Campbell and Tyystjärvi, 2012).

$$\frac{Y_{t}}{Y_{0}}=\frac{K_{rec}}{K_{\mathrm{pi}}+K_{rec}}+\frac{K_{\mathrm{pi}}}{K_{\mathrm{pi}}+K_{rec}}\;e^{-\left(K_{\mathrm{pi}}+K_{rec}\right)t}$$

Where *Y_t_* and *Y_0_* represent the maximal quantum yields at time t and time zero (in seconds), respectively.

### 2.4 Immunoblotting for PsbA protein

Samples (20 mL) were vacuum-filtered onto glass-fiber filters (GF/F, diameter, 25 mm; Whatman), frozen in liquid nitrogen, and kept at −80°C before analysis. The total protein was quantified by the Lowry method using a kit (Bio-Rad) before probing for PsbA. Samples of total protein (1 μg) were separated on 6–12% SDS-PAGE gels (60 min, 200 V) before transfer to PVDF membranes which were probed with a primary antibody recognizing the C-terminal region of PsbA (Agrisera, 1:50,000) (Xu et al., 2019) and an HRP-coupled anti-rabbit secondary antibody. Tanno Gis Analyzer software (Tanno 5,200, China) was used for scanning and analysis of the blots. The removal rate of PsbA (*K_PsbA_*, s^−1^) was determined as described (Gao et al., 2018a,b).

### 2.5 Determination of catalase (CAT) and superoxide dismutase (SOD) activities

Twenty milliliters of sample were collected onto a polycarbonate membrane (0.22 μm, Whatman) and rinsed with phosphate buffer (pH 7.6). Proteins were extracted in 600 μL of the same buffer containing 1 mM EDTA, 50 mM KH_2_PO_4_, and 1% (w/v) polyvinylpolypyrrolidone followed by centrifugation (12,000 × g, 10 min, 4°C). CAT activity was determined using a kit (Nanjing Jiancheng Biological Engineering Company, China) by examining the initial disappearance rate of hydrogen peroxide (H_2_O_2_) at 240 nm (Li et al., 2015). SOD was also measured using a kit (Nanjing Jiancheng Biological Engineering Company, China) with the enzyme concentration causing 50% dismutation of the superoxide radical corresponding to one unit (De la Rosa et al., 2021).

### 2.6 Microcystins (MCs) measurement

The samples were collected and centrifuged (10,000 × g, 10 min, 4°C). The pellets were resuspended in PBS and subjected to freezing and thawing (liquid nitrogen and 4°C, respectively) to induce cell breakage. After re-centrifugation (10,000 × g, 10 min, 4°C), the supernatants were diluted with Milli-Q water and the MCs contents measured by ELISA with a QuantiPlate™ Kit for Microcystin (EnviroLogix, USA), following the provided directions, reading absorbances at 450 nm with a multimode reader (BioTek, USA). The validation of ELISA has been demonstrated to exhibit a high correlation of over 99% with high-performance liquid chromatography, as evidenced by Sheng et al. (2007).

### 2.7 Statistical analysis

SPSS v. 22.0 was used for data analysis. The effects of light exposure times on *F_v_/F_m_*, NPQs, PsbA and CAT and SOD activities were evaluated by ANOVAs (RM-ANOVAs), while the effects of temperature, UVR, and lincomycin were examined using multivariate analysis of variance (MANOVA), followed by Tukey’s HSD tests. Two-way ANOVAs were used for assessing the influence of temperature and UVR on *K_PsbA_*, *K_pi_* and *K_rec_*, while one-way ANOVAs were used to assessing MCs differences. *p* < 0.05 was considered statistically significant.

## 3 Results

**Figure 1:**
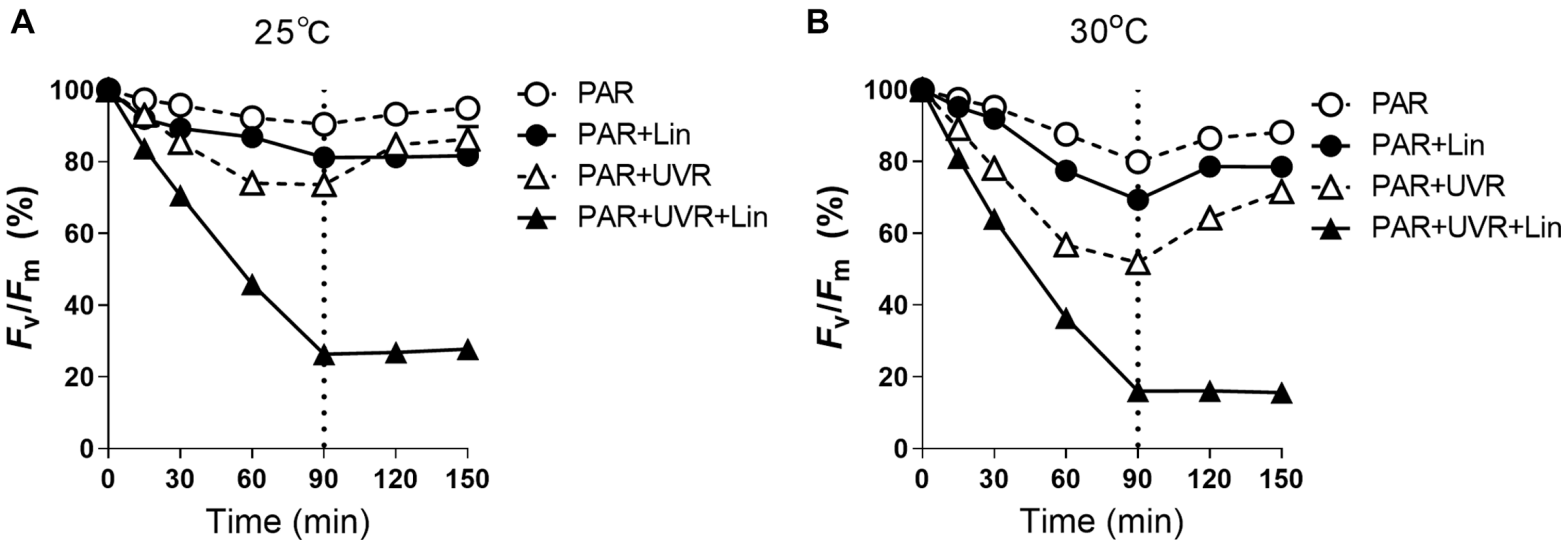
The maximum photochemical yield (*F_v_/F_m_*) changes in *Microcystis aeruginosa* FACHB 905 treated without or with lincomycin (+Lin). Cultures grown at 25°C **(A)** or 30°C **(B)** were exposed to PAR (40.8 Wm^−2^) and PAR + UVR (40.8 Wm^−2^ + 5.0 Wm^−2^) for 90 min, and then shifted to PAR (8.2 Wm^−2^) for 60 min. The dotted line indicates the division between light exposure and recovery period. Data are the means ± SE (*n* = 4), most error bars within symbols.

When *M. aeruginosa* cells cultured at different temperature were treated with PAR and PAR + UVR, the PSII photochemical yield (*F_v_/F_m_*) decreased in correspondence with the duration of exposure (*p <* 0.05) (Figure 1). After 90 min, the *F_v_/F_m_* of the cells growing at 25°C decreased to about 90.5 and 73.6% of the initial values for the PAR and PAR + UVR treatments, respectively (Figure 1A), while at 30°C, the reduction in the *F_v_/F_m_* value was larger, being 80.0% for PAR and 52.0% for PAR + UVR (Figure 1B). Cells exposed to lincomycin showed more marked decreases in *F_v_/F_m_* over the 90-min period at both temperatures (*p <* 0.05), especially in the case of UVR treatment where *F_v_/F_m_* was reduced to 26.3% at 25°C and 16.0% at 30°C of the initial values. Following transfer to low-light conditions for recovery, the *F_v_/F_m_* returned to 88.2–94.9% of the time-0 value at both temperatures when lincomycin was absent (*p <* 0.05). After UVR treatment, cells recovered more slowly, with values of 86.3% at 25°C and 71.7% at 30°C. The cells treated with lincomycin, however, did not recover (Figure 1).

The rate constants for PSII repair (*K_rec_*, s^−1^) and photoinactivation (*K_pi_*, s^−1^) are shown in Table 1. The rise of temperature provoked higher *K_pi_*, while exposure to UVR resulted in a marked increase in *K_pi_* to approximately five times than that of PAR (*p <* 0.05). Interactions between temperature and UVR were more obvious in the case of *K_rec_* (*p <* 0.05), where higher values resulted from UVR treatment relative to those of PAR at 25°C, while only temperature or temperature combined with UVR significantly lowed the *K_rec_* at 30°C compared with that at 25°C (*p <* 0.05).

**Table 1:** The rate constant for PSII repair (*K_rec_*, s^−1^) and photoinactivation (*K_pi_*, s^−1^) and the ratio of *K_rec_* to *K_pi_* for various treatments.

Temperature	Radiation treatments	*K_rec_*	SE	*K_pi_*	SE	*K_rec_*/*K_pi_*
25°C	PAR	0.000371^c^	0.000025	0.000042^a^	0.000003	8.83
	PAR + UVR	0.000670^d^	0.000057	0.000220^b^	0.000006	3.05
30°C	PAR	0.000235^a^	0.000027	0.000066^c^	0.000002	3.64
	PAR + UVR	0.000309^b^	0.000037	0.000282^d^	0.000010	1.10

Data are the means ± SE of four replication. Superscripts with different letters represent significant differences (*p* < 0.05) among treatments.

**Figure 2:**
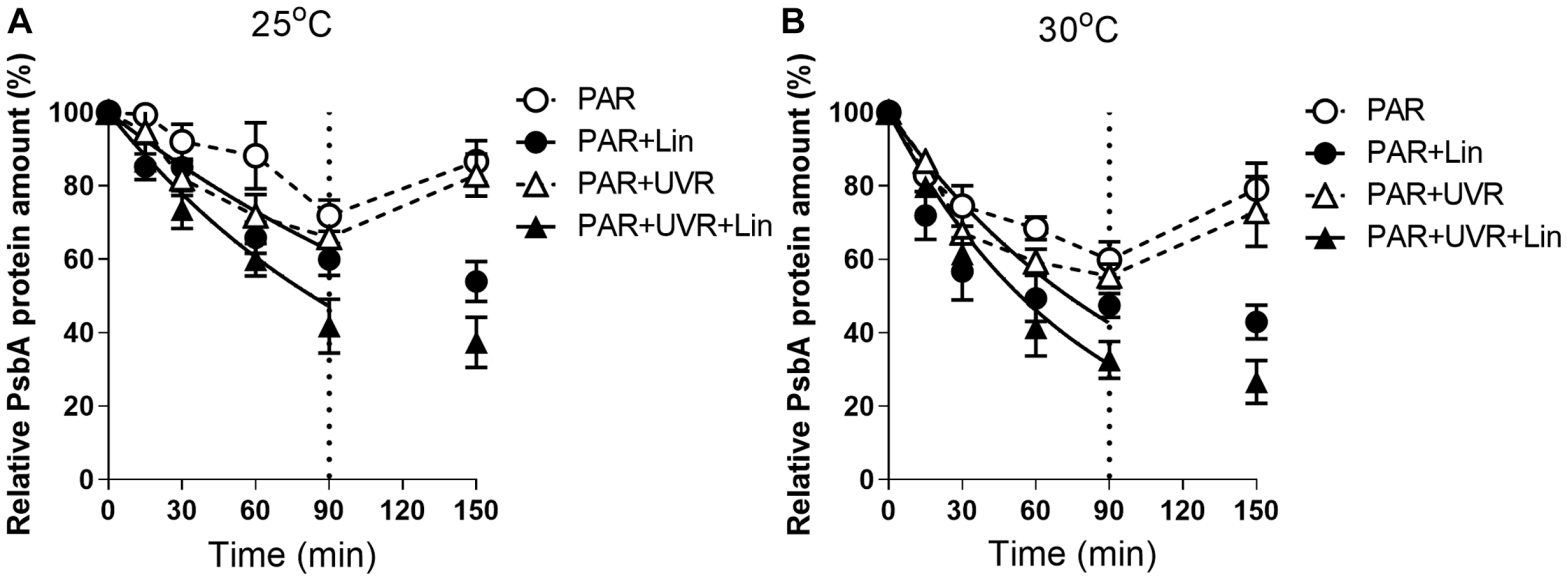
PsbA content changes in *M. aeruginosa* FACHB 905 treated without or with lincomycin (+Lin). Cultures grown at 25°C **(A)** or 30°C **(B)** were exposed to PAR (40.8 Wm^−2^) and PAR + UVR (40.8 Wm^−2^ + 5.0 Wm^−2^) for 90 min, and then shifted to PAR (8.2 Wm^−2^) for 60 min. The dotted line indicates the division between light exposure and recovery period. Data are the means ± SE (*n* = 4).

Growth of cultures with PAR and PAR + UVR led to marked reductions in the PsbA levels as the treatment duration increased (*p <* 0.05) (Figure 2). After 90 min of radiation treatment, both the UVR (*p <* 0.05) and the temperature (*p <* 0.05) markedly altered the PsbA content, but there was no interaction between them (*p =* 0.666) (Figure 2). PsbA content decreased to 71.9 and 65.9% of the initial values after 90 min of PAR or PAR+ UVR treatment at 25°C respectively, while 57.4 and 55.4% at 30°C (*p <* 0.05). After culture with lincomycin, cells contained markedly lower concentrations of PsbA irrespective of temperature or UVR changes (*p <* 0.05) (Figure 2). However, after the restoration under growth light, the PsbA content rose to about 85% at 25°C and 76% at 30°C compared with the initial for both radiation treatments, while cells treated with lincomycin did not recover.

**Figure 3:**
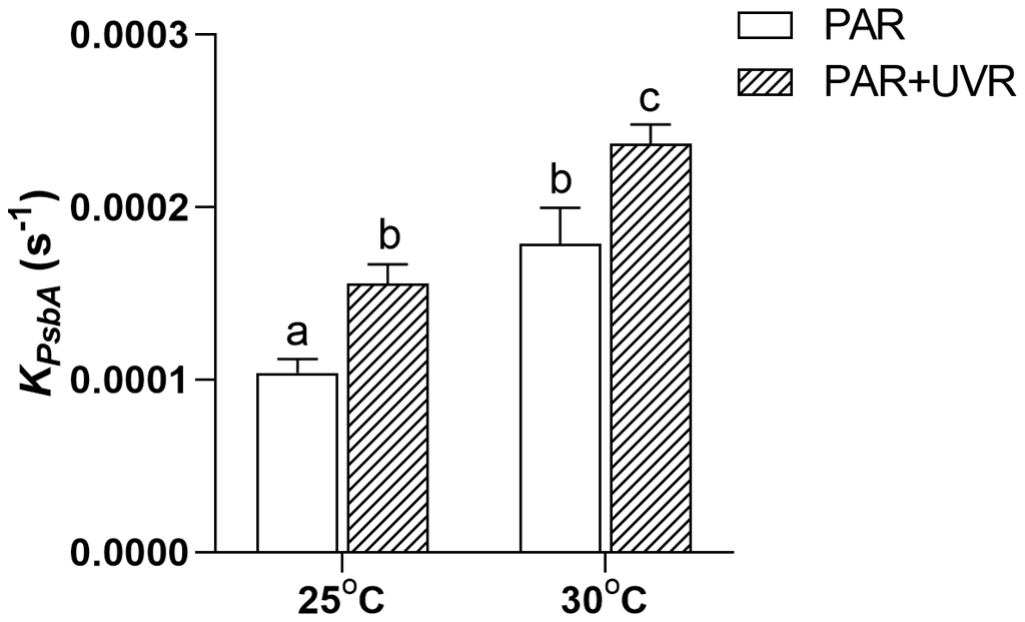
PsbA protein removal rate constant (*K_PsbA_*, s^−1^) in *M. aeruginosa* FACHB 905 grown at 25°C or 30°C. Data are the means ± SE (n = 4). Different letters above error bars indicate significant differences (Tukey HSD) (*p* < 0.05) among treatments.

UVR significantly increased the rate of PsbA removal (*K*_*PsbA*,_ s^−1^) in *M. aeruginosa* FACHB 905 (*p <* 0.05) at both temperatures (Figure 3), the rise of temperature alone or in combination with UVR further promoted the removal rate (*p* < 0.05).

**Figure 4:**
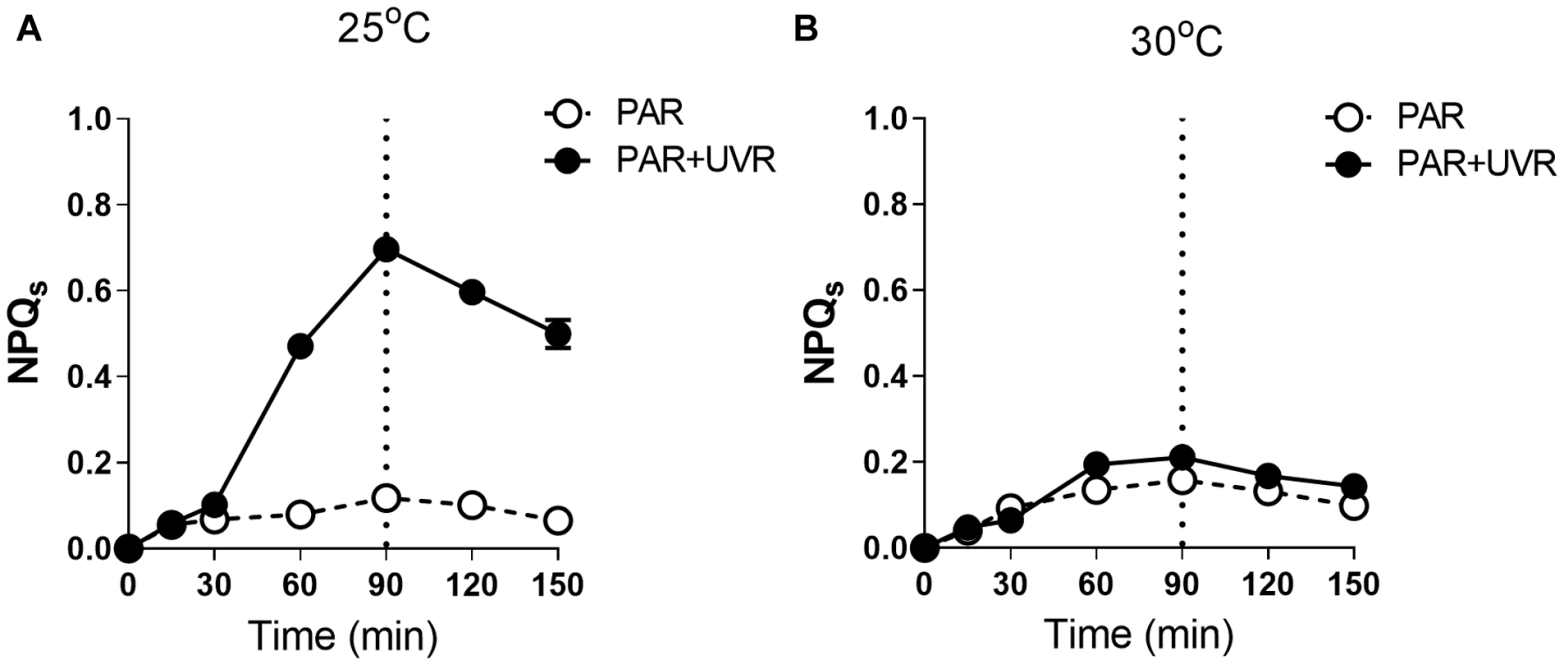
The sustained NPQ (NPQs) changes in *M. aeruginosa* FACHB 905 grown at 25°C **(A)** or 30°C **(B)**. Cultures were exposed to PAR (40.8 Wm^−2^) and PAR + UVR (40.8 Wm^−2^ + 5.0 Wm^−2^) for 90 min, and then shifted to PAR (8.2 Wm^−2^) for 60 min. The dotted line indicates the division between light exposure and recovery period. Data are the means ± SE (*n* = 4).

NPQs was induced sharply during the radiation exposure with PAR + UVR at 25°C (*p <* 0.05), while only a slight increase showed under PAR exposure (Figure 4A) The rise of temperature to 30°C decreased the induction of NPQs under both PAR and PAR + UVR exposure with no significant difference between them (Figure 4B *p >* 0.05). After recovery, the NPQs relaxed irrespective of the radiation treatments.

**Figure 5:**
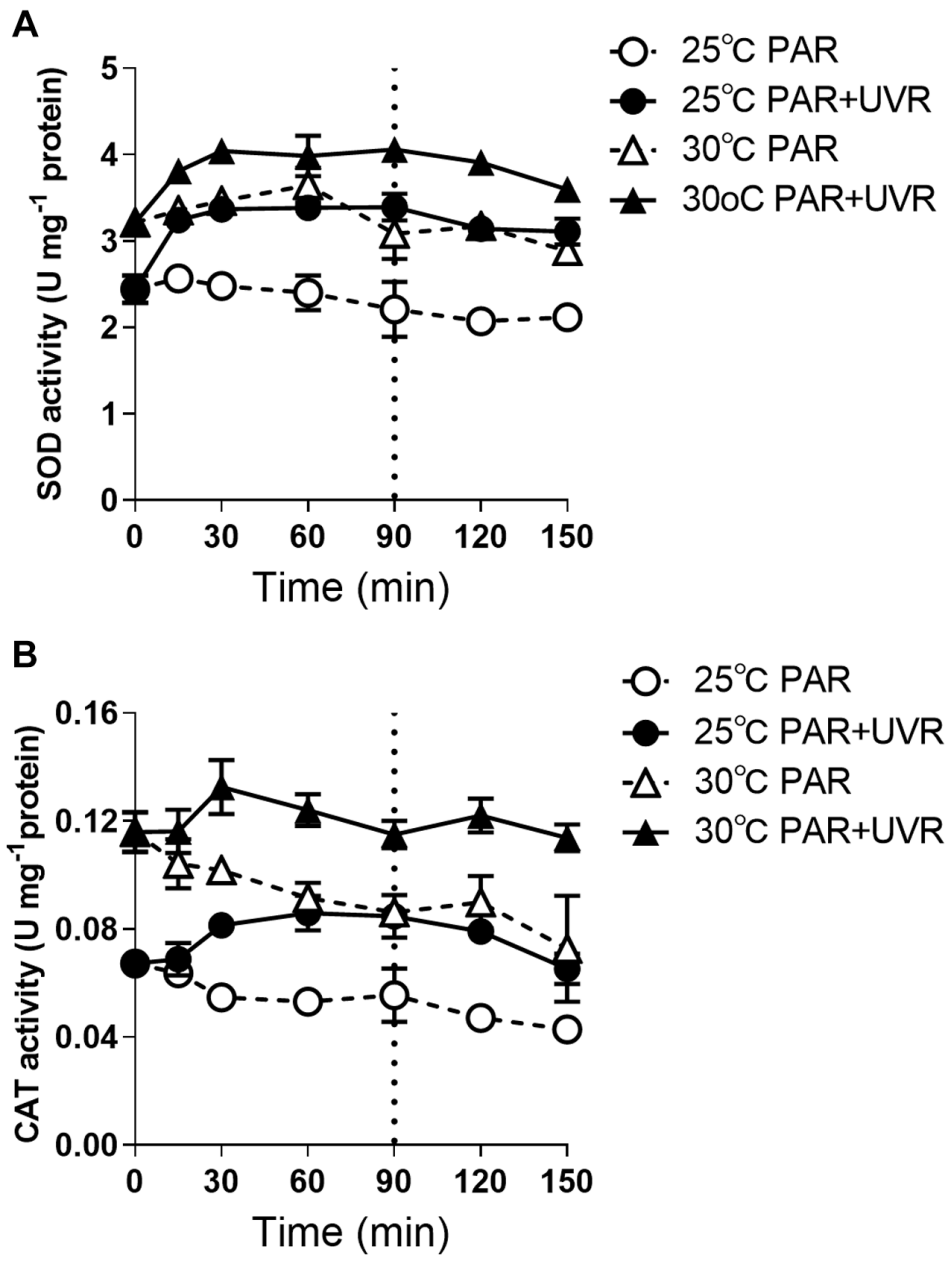
SOD **(A)** and CAT **(B)** activity changes in *M. aeruginosa* FACHB 905 grown at 25°C or 30°C. Cultures were exposed to PAR (40.8 Wm^−2^) and PAR + UVR (40.8 Wm^−2^ + 5.0 Wm^−2^) for 90 min, and then shifted to PAR (8.2 Wm^−2^) for 60 min. The dotted line indicates the division between light exposure and recovery period. Data are the means ± SE (*n* = 4).

Cells grown at 30°C had higher activities of both CAT and SOD before the radiation treatment than those at 25°C (Figure 5, *p <* 0.05). Both activities remained stable during exposure to PAR except the CAT activity in cells exposed to PAR at 30°C, which decreased to 73% of the initial value at time 0 (*p <* 0.05). In contrast, PAR + UVR exposure markedly elevated both SOD (*p <* 0.05) and CAT (*p <* 0.05) activities at both temperatures.

**Figure 6:**
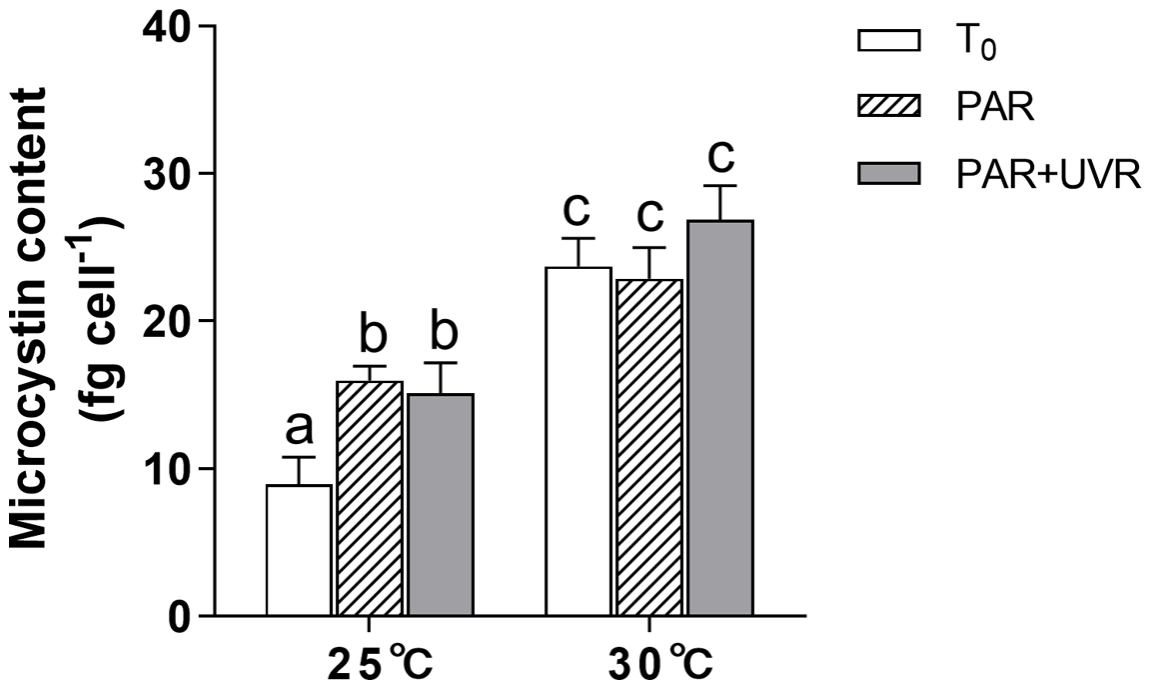
Changes of the microcystin content in *M. aeruginosa* FACHB 905 grown at 25°C or 30°C before (T_0_) and after 90 min-exposure with PAR (40.8 Wm^−2^) and PAR + UVR (40.8 Wm^−2^ + 5.0 Wm^−2^). Data are the means ± SE (*n* = 4).

MCs content increased obviously after the exposure of PAR and PAR + UVR at 25°C (Figure 6); the difference between the two was non-significant (*p >* 0.05). Cells grown at 30°C showed higher MCs content than those at 25°C regardless of the radiation treatments. It showed that increased temperature exhibited the main effect on MCs production (*p <* 0.05).

## 4 Discussion

This study showed that *M. aeruginosa* FACHB 905 was more resistant to both PAR and PAR + UVR exposure at growth temperature (25°C) as indicated by the less inhibition on PSII quantum yield (*F_v_/F_m_*) in comparison with that at increased temperature of 30°C, as expected, the presence of UVR significantly exacerbated the photoinhibition, and the increased temperature worked synergistically with UVR leading to a further inhibition on *F_v_/F_m_*. To explore the underlying mechanism, we evaluated the alterations in PsbA content to evaluate the turnover of subunits and recovery of PSII function. The function of PSII often drops more rapidly than the content of PsbA, suggesting that the photoinactivated but intact PbsA may accumulate (Wu et al., 2011). However, here, it was found that the reductions in PSII activity coincided approximately with decreases in the PsbA levels for the samples exposed to PAR + UVR at high temperature, and in other treatments PSII function dropped slower than PsbA content. The different correspondences between PsbA levels and PSII function in *M. aeruginosa* FACHB 905 suggest the possibility of a regulatory adaptation (Edelman and Mattoo, 2008; Wu et al., 2011), in which radiation treatment triggers increased PsbA clearance from accumulated intact but photoinactivated PSII pools in the presence of low growth light (Gao et al., 2018a,b). This removal of PsbA from photoinactivated PSII centers was increased after exposure to UVR and elevated temperature (Figure 3), suggesting a possible protective mechanism against these forms of stress (Xu et al., 2023), as rapid clearance of defective PsbA may lead to the incorporation of fresh PsbA proteins, as has been reported in several diatoms (Zang et al., 2022; Xu et al., 2023). However, different from diatoms, cyanobacteria possess a small gene family coding for the various, functionally distinct PsbA isoforms (Mulo et al., 2012). For example, in *Synechococcus* PCC7942, under environmental stresses, including reduced temperature, increased light or UV-B, the expression of *psbA* shifts to the replacement of the PsbA:1isoform (encoded by *psbA1*) with PsbA:2 (encoded by *psbA2* and *psbA3)* (Kós et al., 2008). When PSII centers contains PsbA:2, cells showed increased resistance against UV radiation (Campbell et al., 1998). This could partly contribute to the relatively higher *K_rec_* values in the presence of UVR, particularly for cells grown at 25°C. In contrast, the PsbA protein isoform exchange might not occur at high temperature in *M. aeruginosa,* as found in the cyanobacterium *Thermosynechococcus elongates* BP-1 and *Synechococcus* sp. PCC 7942 (Campbell et al., 1996; Kós et al., 2008), thus we assumed that the antagonistic effect between high temperature and radiation treatments on the regulation of PsbA protein isoform exchange might somehow account for the lower *K_rec_* value at 30°C. However, the turnover of PsbA isoforms in this study regardless of the treatment conditions was insufficient to compensate for the progressively declination of PSII function as shown in Figure 1.

Both temperature and UVR stress can lead to increased production of ROS, potentially generating oxidative stress. The antioxidant enzyme SOD responds rapidly by catalyzing the conversion of superoxide radicals into H_2_O_2_ that is then converted to H_2_O by CAT (Rastogi et al., 2014; Cho et al., 2021). In this study, cells grown at elevated temperature showed higher SOD and CAT activity than that at 25°C, and the subsequent exposure with UVR further stimulated the activity of both antioxidases, indicating their protective role in mitigating harmful effects of oxidative stress caused by UVR. Both UVR and increases in temperature are known to induce increased production of these enzymes in *Anabaena* sp. and *M. aeruginosa* CAAT 2005–3 (Singh et al., 2013; De la Rosa et al., 2021). However, the response in *M. aeruginosa* did not appear to be sufficient to counteract ROS entirely as there was still photosynthetic activity along with reduced PsbA. On the other hand, elevated temperature and UVR may increase the membrane peroxidation by triggering the up-regulation of ROS and destabilization of membranes (Mittler et al., 2012), thus probably affecting the formation of NPQ, since cyanobacteria possess a medium NPQ quenching component, i.e., state transition process (qT), associated with thylakoid membrane fluidity (Wang et al., 2012). It has been found that *M. aeruginosa* strains may have a fast NPQ component, resulting from high-light conditions, derived from conformational alterations in photosynthetic pigment proteins in the thylakoid membrane (PPPTM) (Wang et al., 2012). Thus, it is possible that UVR may alter the conformations of PPPTMs, inducing NPQ at normal growth temperatures (Xu et al., 2019), while less NPQ was formed due to the elevated temperature and together with the outweighted UVR damage effects.

MCs have been reported to act as protein-modulating metabolites and protectants, thus increasing the fitness of MCs producers under stress conditions (Zilliges et al., 2011). In this study, both PAR and PAR + UVR treatment markedly increased MCs synthesis at growth temperature of 25°C, indicating their protective role against oxidative stress. In addition, elevated temperature provoked further production of MCs, this result aligns with the previous research that the rise of culture temperature from 20°C to 30°C induced a marked elevation in the expression of one of the MCs genes, *mcyB* (Scherer et al., 2017). However, MCs production may be energetically costly (Kardinaal et al., 2007a,b; Lei et al., 2015), reducing the availability of resources for other functions, for example, PsbA synthesis (Xu et al., 2019), thus probably causing the lower *K_rec_* at high temperature.

## 5 Conclusion

In this study, changes in physiological parameters features and PSII repair in *M. aeruginosa* grown at higher temperatures with UV radiation were investigated. Increased temperature was observed to enhance the sensitivity of *M. aeruginosa* to UV radiation mainly through reducing the PSII repair rate and depressing the NPQs induction. Although elevated temperature itself or in combination with UV radiation induced higher SOD and CAT activity and promoted the removal rate of PsbA, these did not appear sufficient to counter their negative effects. In addition, high temperature may favor the preferential synthesis of MCs, or this energetically costly process might lead to the less energy and materials allocated to the synthesis of PsbA, which partly accounting for the lower *K_rec_* and the declination of photosynthetic activity of cells exposed to PAR and PAR + UVR. These findings suggest that high temperature reduced the adaptability of *M. aeruginosa* to UVR stress, which would affect its competitiveness and abundance in water environments of the future.

## Data availability statement

The original contributions presented in the study are included in the article/supplementary material, further inquiries can be directed to the corresponding author.

## Author contributions

FY: Conceptualization, Writing – original draft. ML: Conceptualization, Methodology, Writing – review & editing. SZ: Data curation, Formal analysis, Writing – review & editing. ZX: Formal analysis, Funding acquisition, Investigation, Writing – review & editing. MB: Formal analysis, Funding acquisition, Writing – review & editing. HW: Conceptualization, Funding acquisition, Project administration, Writing – review & editing.
